# Compliance with the Australian 24-hour movement guidelines for the early years: associations with weight status

**DOI:** 10.1186/s12889-017-4857-8

**Published:** 2017-11-20

**Authors:** Rute Santos, Zhiguang Zhang, João R. Pereira, Eduarda Sousa-Sá, Dylan P. Cliff, Anthony D. Okely

**Affiliations:** 1 Early Start, Faculty of Social Sciences, University of Wollongong, Wollongong, NSW 2522 Australia; 2 Research Centre in Physical Activity, Health and Leisure, Faculty of Sport, University of Porto, Porto, Portugal; 3 Research Centre in Sport and Physical Activity, Faculty of Sport Science and Physical Education, University of Coimbra, Coimbra, Portugal; 4 Illawarra Health and Medical Research Institute, University of Wollongong, Wollongong, Australia

**Keywords:** Adiposity, Body mass index, Recommendations, Physical activity, Sedentary behavior, Childhood, Screen time, Sleep

## Abstract

**Background:**

For effective public health and surveillance it is important to document the proportion of young children who meet the new Australian Integrated 24 h Movement Guidelines for the Early Years and how these associate with health outcomes. We aimed to (i) assess compliance with the new Integrated 24 h Movement Guidelines for the Early Years in a sample of Australian toddlers; and (ii) ascertain whether compliance with the guidelines associates with weight status.

**Methods:**

The sample comprised 202 toddlers (104 girls) aged 19.74 ± 4.07 months from the GET UP! Study. Participants wore accelerometers (Actigraph GT3X+) for 24 h over 7 consecutive days to assess physical activity, sedentary time and sleep. Parents reported participants’ screen time. Weight and height were measured and body mass index (BMI) z-scores by age and sex were calculated. Analysis of Covariance (ANCOVA) was performed to test differences in BMI z-scores between participants complying with (i) none or any individual guideline, (ii) any combination of meeting two guidelines, and (iii) those who met all three guidelines, adjusting for child age, gender and socioeconomic status.

**Results:**

Only 8.9% of the sample met the overall 24 h movement guidelines. Most of the sample met the physical activity (96.5%) and sleep (79.7%) guidelines but only 11.4% met the sedentary behavior guideline. Average BMI Z-scores did not significantly differ between children who complied with none or any individual guideline, any combination of meeting two guidelines, and those who met all three guidelines (*p* > 0.05). Although the lack of significant differences, participants who accomplished any combination of two guidelines or all three guidelines appear to have had a lower BMI Z-score than those complying with one of the guidelines or none.

**Conclusions:**

Just under 9% of our sample met the overall Australian 24 h Movement Guidelines for the Early Years. BMI was not associated with the accomplishment of any of the 24-h Movement Guidelines. Strategies to promote adherence to the 24-h movement guidelines in toddlers, particularly for screen time, are necessary, as promoting health-related behaviors in early childhood has the potential to provide children a strong foundation for lifelong physical and mental health.

**Electronic supplementary material:**

The online version of this article (10.1186/s12889-017-4857-8) contains supplementary material, which is available to authorized users.

## Background

Overweight and obesity during childhood are major public health concerns. Globally it is estimated that 6.7% of children under the age of 5 are overweight or obese and it is expected that this prevalence will reach 9.1% by 2020 [1]. In Australia, the prevalence of overweight and obesity in pediatric ages has increased substantially in the last two decades, particularly among those from low socio-economic backgrounds [2, 3]. Although the prevalence has leveled off somewhat over the past decade [2, 3], there are still 1 in 5 Australian children aged 2 to 4 years who are overweight or obese [4]. These figures are particularly disturbing given the plethora of short term and long-term adverse health consequences associated with childhood overweight and obesity [5, 6].

From a movement perspective, sleep, sedentary behavior and physical activity represent a continuous range of movement (from none/low to high) with a daily time allocation of these behaviors representing portions of a 24-h period [7, 8]. The new evidence-based Canadian and Australian Integrated 24-h Movement Guidelines for the Early Years acknowledge this movement continuum over the 24-h period and provide guidance for each of the behaviors that compose the whole day: sleep, sedentary behavior and physical activity [9]. For effective public health and surveillance, it is important to document the proportion of young children meeting each of these guidelines as well their combination and how these associate with health outcomes.

Studies have shown that time spent in each of these behaviors generally associate with early childhood adiposity, despite some mixed findings [10–16]. However, there are only a few studies with toddlers looking at the relationship between each of movement behaviors and their combination with adiposity [13–16].

In this context, the aims of this study were to (i) assess compliance with the new Integrated 24-h Movement Guidelines for the Early Years in a sample of Australian toddlers; and (ii) ascertain whether compliance with the guidelines associates with weight status.

## Methods

### Study design and sampling

This paper reports on a cross-sectional analysis of the baseline data from the *Get Up! Study*, a cluster randomized controlled trial aimed to evaluate the effects of reduced sitting on toddlers’ cognitive development. Detailed description of sampling, rationale and data collection procedures of this study are described elsewhere [17]. Briefly, baseline data were collected in 2016 in 335 apparently healthy toddlers (155 girls), i.e. without any medication or medical diagnosis of physical or mental impairment, aged 19.8 ± 4.08 months; of these, 284 had valid accelerometer data and 202 had data on screen time. Therefore, the final sample for this paper comprises 202 toddlers (aged 12 to 28 months).

The *Get Up! Study* was conducted in accordance with the Helsinki Declaration for Human Studies and approved by the University of Wollongong’s Human Research Ethics Committee (HE15/236) and the RCT was registered in the Australian and New Zealand Clinical Trials Registry (ACTRN12616000471482, 11/04/ 2016, retrospectively registered). Informed written consent was obtained from children’s parents or guardians.

### Measures

#### Physical activity, sedentary time and sleep

Physical activity, sedentary time and sleep over a usual week were measured with Actigraph GT3X+ accelerometers. These accelerometers have established validity and utility in toddlers [18, 19]. Participants were asked to wear the accelerometer, attached tightly on the right hip, for 24 h/day over 7 consecutive days and parents and educators were asked to register, in an activity monitor log, the times that the accelerometer was removed from the child, as well as the naps and night sleep hours. Data were collected using a sampling rate of 30 Hz and then reintegrated into 15 s epoch for analysis.

Considering the accelerometers logs, data files from individual participants were manually and visually screened to detect naps, nighttime sleep periods and non-wear time. Participants had to have at least 1 day (i.e. one period of 24-h) with accelerometer data to be included in the analyses. After screening was completed, the raw activity ‘counts’ were processed for determining the time spent in sedentary behaviors and different physical activity intensities.

Accelerometer cut-points for toddlers were used as follows: sedentary time < 25 counts/15 s; 25–420 counts/15 s; moderate to vigorous physical activity (MVPA) > 420 counts/15 s [18]. A bout of sedentary time was defined as a period of time that was maintained completely at an intensity level of less than < 25 counts/15 s, for one hour or longer. Accelerometer data were analyzed using an automated data reduction program (ActiLife Software, version 6.12.1 for Windows).

### Body mass index

Body height was measured to the nearest 0.1 cm in bare or stocking feet with the child standing upright against a portable stadiometer (Seca 254 Hamburg, Germany). Body weight was measured to the nearest 0.10 kg, with the child lightly dressed (and without diapers) using a portable electronic weight scale (Seca 254 Hamburg, Germany). Body mass index (BMI) was calculated as weight(kg)/ height(m)^2^. BMI Z-scores by age and sex were calculated. Participants were classified as underweight, normal weight, overweight or obese according to the World Health Organization age and sex specific criteria [20].

### Screen time

Parents were asked to report the child’s screen time by answering the following questions: “For how long does your child use screen entertainment on a typical weekday?” and “For how long does your child use screen entertainment on a typical weekend day?” Time reported in both questions was summed as follows: (screen time weekdays * 5 + screen time weekend days *2) / 7 = average screen time per day.

### Socio-economic status

Family socio-economic status was assessed using the family postcode address as a proxy-measure of the child socio-economic status, according to the Australian Socio-Economic Indexes for Areas 2011 (SEIFA-Index of Relative Socio-Economic Disadvantage) [21].

### Operational definitions of the integrated 24 h movement guidelines

#### Physical activity

Participants were classified as meeting the physical activity guideline if they averaged at least 180 min per day of physical activity of any intensity (i.e., light, moderate or vigorous intensity physical activity), including at least 1 min of MVPA, as the guidelines for physical activity include “some energetic play” within these 180 min of physical activity.

#### Sedentary behavior

Participants were classified as meeting the sedentary behavior guideline if they had concurrently (i) no bouts of sedentary time lasting one or more hours; and (ii) no screen time (for those aged ≤ 24 months) or less than 1 h/day of screen time (for those aged > 24 months).

#### Sleep

Participants were classified as meeting the sleep guideline if their average sleep duration was between 11 and 14 h per 24-h, including nap(s) and night time sleep.

### Statistics

Descriptive characteristics are presented as means and standard deviations (SD) for continuous variables and as percentages for categorical variables. Two-tailed Student t-tests or Mann-Whitney U Test and Chi-square tests were performed to assess gender differences for continuous and categorical variables, respectively.

For descriptive purposes only, consistency of bedtime and wake up time was calculated as the intra-subject standard deviation of the average bedtime and wake up time of each participants that had at least 2 days of accelerometer data (*n* = 173).

Analysis of Covariance (ANCOVA) was performed to test differences in BMI z-scores between participants complying with (i) none or any individual guideline, (ii) any combination of meeting two guidelines, and (iii) those who met all three guidelines, adjusting for child age, gender and socioeconomic status.

A 0.05 level of statistical significance was considered. Data analysis was performed using IBM SPSS®, version 24.0 (SPSS Inc., Chicago, IL, USA).

## Results

Descriptive characteristics of the participants are presented in Table 1. Boys had on average less sedentary time than girls (*p* < 0.05). None of the participants had sedentary bouts of 1 h or more. The percentage of overweight and obese participants was 22.8 and 4%, respectively. Regarding accelerometer wear time, 14.4% of participants had 1 day (i.e. one 24-h period) of accelerometer data, 4% had 2 days and 81.6% had 3 or more days of accelerometer data; and children were monitored on average for 5.14 ± 2.27 days. Average accelerometer wear time was 1297.4 ± 93.0 min per day.

**Table 1:** Participants Characteristics (mean ± standard deviation)

	Total Sample (*n* = 202)	Girls (*n* = 104)	Boys (*n* = 98)	*p* ^c^
Age (months)	19.74 ± 4.072	19.76 ± 4.05	19.71 ± 4.12	0.884
BMI (kg/m^2^)	17.95 ± 1.64	17.78 ± 1.71	18.14 ± 1.56	0.427
BMI z-scores	0.07 ± 0.93	0.06 ± 0.98	0.08 ± 0.89	0.934
Total physical activity (min/day)	294.97 ± 59.03	289.98 ± 57.92	300.27 ± 60.02	0.109
Light physical activity (min/day)	237.65 ± 44.92	235.95 ± 45.14	239.45 ± 44.86	0.351
Moderate to vigorous physical activity (min/day)	57.32 ± 21.40	54.03 ± 18.42	60.82 ± 23.77	0.055
Sedentary time (min/day)	262.77 ± 60.16	271.18 ± 59.97	253.85 ± 59.37	**0.025**
Screen time (min/day)	76.38 ± 62.81	77.71 ± 71.98	74.97 ± 51.66	0.557
Sleep time (min/day)^a^	739.66 ± 73.96	731.18 ± 72.25	784.66 ± 75.05	0.126
Consistency of bedtime (standard deviation of average bedtime in min)^b^	44.71 ± 31.12	42.51 ± 23.42	47.05 ± 37.61	0.907
Consistency of wake up time (standard deviation of average wake up time in min)^b^	45.56 ± 36.42	47.53 ± 45.8	43.5 ± 22.71	0.410
Accelerometer wear time (min/day)	1297.40 ± 93.00	1292.34 ± 95.06	1302.78 ± 90.93	0.355

*BMI* body mass index

^a^including nap(s) time

^b^analysis performed on a subsample of 173 toddlers with at least 2 days of accelerometers data

^c^Compares genders with Two-Tailed T-test (BMI) or Mann Whitney U Test (all other variables

Bold significant difference between boys and girls (*p*<0.05)

**Fig. 1:**
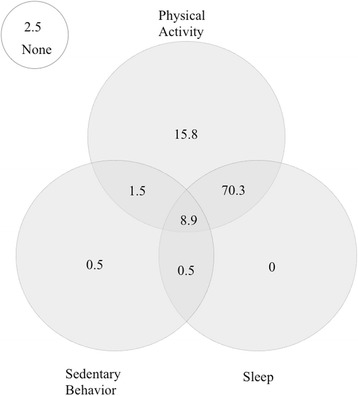
Venn diagram showing the proportion (%) of toddlers meeting no guidelines, physical activity, sedentary behavior, sleep guidelines and the combinations of these guidelines in the full sample (*n* = 202)

Figure 1 shows the proportion (%) of toddlers meeting no guidelines, one guideline and combinations of the three guidelines. Most of the sample (96.5%) met the physical activity guideline, but only 11.4% met the sedentary behavior guideline. Only 2.5% of the sample did not meet any of the guidelines, 16.3% met at least one guideline, 72.3% met at least two guidelines and 8.9% of the sample met all three guidelines. Children that met the physical activity guideline had at least 15.5 min/day of MVPA. The Figure - Additional file 1, shows the proportion (%) of toddlers meting no guidelines, physical activity, sedentary behavior, sleep guidelines and the combinations of these guidelines, separately for girls and boys. No significant differences were found between genders regarding the proportion of toddlers meeting the guidelines (X^2^ = 0.929, *p* = 0.629).

**Fig. 2:**
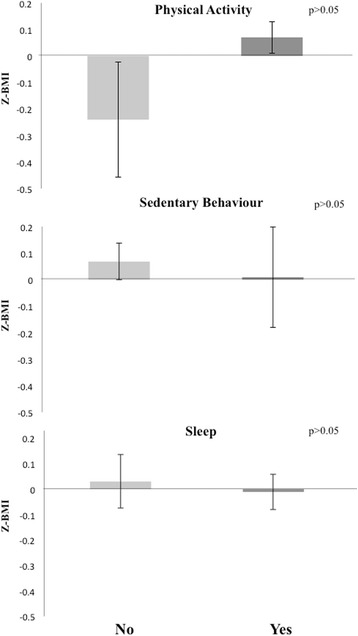
Average BMI Z - score per group according to the accomplishment of each of the 24 – hour Movement Guidelines Bars represent mean values and error bars represent standard errors.

**Fig. 3:**
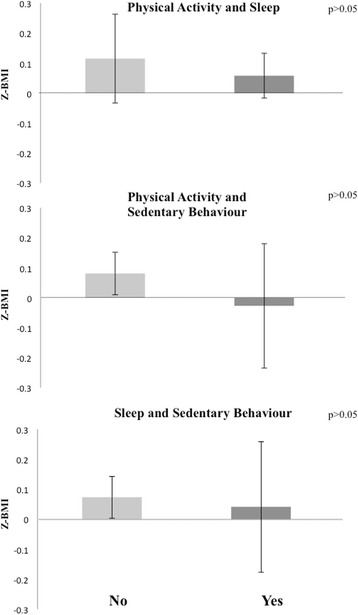
Average BMI Z - score per group according to the accomplishment of combinations of two 24 – hour Movement Guidelines. Bars represent mean values and error bars represent standard errors

**Fig. 4:**
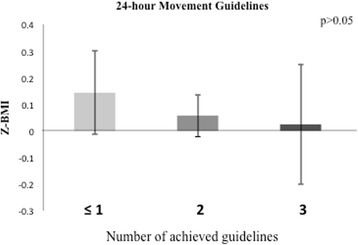
Average BMI Z - score per group according to the accomplishment the 24 – hour Movement Guidelines. Bars represent mean values and error bars represent standard errors

As shown in Figs. 2, 3 and 4 and Additional file 2 average BMI Z-scores did not differ between children who met none or single guidelines, any combination of meeting two guidelines, or all three guidelines (*p* > 0.05).

## Discussion

Using 24-h accelerometer data, we found that just under 10% of toddlers met all three of the current Australian 24 h Movement Guidelines for the Early Years. Nearly the whole sample (96.5%) met the physical activity guideline and only 11.4% met the sedentary behavior guideline. Our results are consistent with those reported for Canadian toddlers and Australian and Canadian preschoolers. The reports of Lee et al. [22], Chaput et al. [23] and Cliff et al. [24], in this supplement, also show that the majority of young children met the physical activity guideline and that 15% or less met all three guidelines of the 24-h Movement Guidelines for the Early Years.

As none of the participants had sedentary bouts of 1 h or more, these 11.4% that met the sedentary behavior guideline reflects the amount of children meeting the screen time component of the sedentary behavior guideline; which is of concern given the potential long-term health impacts of excessive screen-based sedentary behaviors during the first years of life [16]. In our study, girls spent on average more time in sedentary behaviors than boys, a finding that is consistent with previous studies in older children [25–27] but contradicts others [28–31]. In this respect, it is important to notice that the correlates of sedentary behavior appear to be dependent on the type and context of the behavior, as well as how sedentary behavior is assessed [27–33]. Also, as sedentary behaviors seem to track from early childhood to middle childhood [34] and from childhood to adolescence [35, 36] efforts to limit the amount of time spent in sedentary pursuits from an early age are therefore necessary.

The proportion of overweight and obese children in our sample (26.8%) is higher than the most recent data for Australian toddlers and pre-schoolers (22.8%) [4], most likely because our study was conducted with children living in low socio-economic areas. Average BMI Z-scores did not differ significantly between children who complied with none or any individual guideline, any combination of meeting two guidelines, and those who met all three guidelines (*p* > 0.05). Although the differences were not statistically significant, participants who accomplished any combination of two guidelines or all three guidelines appear to have a lower BMI Z-score, than those complying with one of the guidelines or none. These results are in line with the systematic reviews presented in this supplement, where in children aged 0 to 4 years, the associations between adiposity and sedentary time, screen-based sedentary behaviors, shorter sleep duration and lower levels of physical activity, as well as the combination of these were predominantly unfavorable or null [13–16]. Our results also agree with those presented by Lee et al. [22] and Chaput et al. [23] for Canadian toddlers and preschoolers, in this supplement; but contrast with previous findings in older children and adolescents, where such associations were mainly unfavorable [37–40]. Indeed, it seems less likely to find significant associations between the behaviors that are included in the 24-h movement guidelines and adiposity in samples of apparently healthy toddlers, as the deleterious effects of accumulating low physical activity levels, short sleep, higher sedentary time and sedentary screen-based activities on adiposity are likely to manifest over time, as children get older. Nevertheless, our study is unique in the sense that we measured objectively all movement behaviors over a 24-h period with accelerometers (except screen time) and therefore, direct comparisons with previous studies that used different assessments tools, over different periods of the day, including proxy reports, in toddlers, should be made with caution.

Our study is not without limitations. First, we used accelerometer’s cut-points that were developed for slightly older toddlers that those included in our sample (24 months versus 19 months) [18]; and as children’s motor development changes rapidly during the first 2 years of life, the use of this cut-points may have potentially introduced some measurement inaccuracy. Second, our inclusion criteria was limited to 1 day with 24-h of accelerometer data, which may not be representative of a child’s usual movement behavior; however, the majority of our sample (81.6%) had at least 3 days of accelerometer wear time and children were monitored on average for 5 days, which is consistent with previous data in older children [41]. Other limitations include the cross-sectional design of our study, which precludes establishing causality and the fact that we did not consider other potential confounders in our analysis, such as dietary intake. Finally, our sample is not nationally representative and therefore our results may not generalize to the Australian population.

As prevalence estimates affect public health policies and interventions, it is paramount that the prevalence rates are measured as accurately as possible. In this sense, the use of 24-h accelerometer data is the greatest strength of this study.

## Conclusions

Using 24 h accelerometer data, we found that just under 9% of our sample of toddlers met all three of the current Australian 24-h Movement Guidelines for the Early Years, which was mainly due to the low compliance of the sedentary behavior guidelines (11.4%) (i.e. screen time component). The large majority of the sample (96.5%) met the physical activity guideline. BMI was not associated with the accomplishment of the 24-h Movement Guidelines. Further studies with more robust designs and larger samples are necessary to confirm or rule out our findings.

Strategies to promote adherence to the 24-h movement guidelines in toddlers, particularly for screen time, are necessary, as promoting health-related behaviors in early childhood has the potential to provide children a strong foundation for lifelong physical and mental health.

## Additional files

Additional file 1: Mean BMI Z-scores according to level of compliance with the 24 h Movement Guidelines. (DOCX 17 kb)

Additional file 2: Venn diagram showing the proportion (%) of toddlers meeting no guidelines, physical activity, sedentary behavior, sleep guidelines and the combinations of these guidelines for girls (*n* = 104) and boys (*n* = 98). (TIFF 1521 kb)

## Abbreviations

**BMI**: **Body Mass Index**
MVPA: Moderate to vigorous physical activity
SEIFA: Index of relative socio-economic disadvantage
