# Supplementary material for: Compliance with the Australian 24-hour movement guidelines for the early years: associations with weight status
Source: BMC Public Health. 2017 Nov 20;17(Suppl 5):867. doi: 10.1186/s12889-017-4857-8 (PMC5773912; doi:10.1186/s12889-017-4857-8)
Supplement: Supplementary file 1 — Mean BMI Z-scores according to level of compliance with the 24 h Movement Guidelines. (DOCX 17 kb) [file 12889_2017_4857_MOESM1_ESM.docx]

Table S1. Mean BMI Z-scores according to level of compliance with the 24h Movement Guidelines.

|  | **Compliance with guideline** | **BMI Z-Score**  (mean ± SE) | ***F**** | ***p*** |
| --- | --- | --- | --- | --- |
| Physical Activity | No | -0.240 ± 0.218 | 1.329 | 0.250 |
|  | Yes | 0.070 ±0.060 |  |  |
| Sedentary Behaviour | No | 0.068 ± 0.068 | 0.087 | 0.769 |
|  | Yes | 0.009 ± 0.189 |  |  |
| Sleep | No | 0.030 ± 0.105 | 0.105 | 0.746 |
|  | Yes | -0.011 ± 0.070 |  |  |
| Physical Activity  and Sleep | No | 0.115 ± 0.149 | 0.118 | 0.731 |
|  | Yes | 0.058 ± 0.075 |  |  |
| Physical Activity and Sedentary Behaviour | No | 0.081 ± 0.071 | 0.242 | 0.623 |
|  | Yes | -0.027 ± 0.208 |  |  |
| Sleep and Sedentary Behaviour | No | 0.073 ± 0.070 | 0.019 | 0.891 |
|  | Yes | 0.041 ± 0.218 |  |  |
| Guidelines | ≤ 1 | 0.143 ± 0.157 | 0.145 | 0.865 |
|  | 2 | 0.057 ± 0.079 |  |  |
|  | 3 | 0.024 ± 0.225 |  |  |

* ANCOVA – Adjustments for age, gender and socio-economic status.

BMI, Body Mass Index; SE, Standard Error.
