# Supplementary figures and images for: Compliance with the Australian 24-hour movement guidelines for the early years: associations with weight status
Source: BMC Public Health. 2017 Nov 20;17(Suppl 5):867. doi: 10.1186/s12889-017-4857-8 (PMC5773912; doi:10.1186/s12889-017-4857-8)

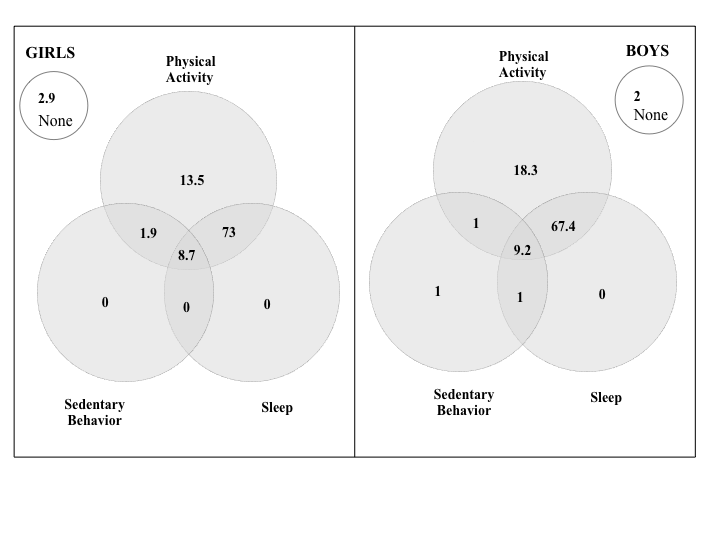

Supplement: Supplementary file 2 — Venn diagram showing the proportion (%) of toddlers meeting no guidelines, physical activity, sedentary behavior, sleep guidelines and the combinations of these guidelines for girls (n = 104) and boys (n = 98). (TIFF 1521 kb) [file 12889_2017_4857_MOESM2_ESM.tiff]
